# Microwave ablation versus resection for small colorectal liver metastases: evolving evidence and clinical considerations

**DOI:** 10.1097/JS9.0000000000005097

**Published:** 2026-03-17

**Authors:** Aime Ishimwe Mugisha

**Affiliations:** College of Medicine and Health Sciences, School of Medicine and Pharmacy, University of Rwanda, Kigali, Rwanda

*Dear Editor,*

The management of small solitary colorectal liver metastases (CRLM) continues to evolve, with minimally invasive strategies increasingly complementing traditional liver resection (LR). Laparoscopic microwave ablation (MWA) has emerged as a promising alternative, offering reduced perioperative morbidity and shorter hospital stay while maintaining favorable long-term oncologic outcomes.

Evidence from recent multicenter studies demonstrates that MWA can achieve comparable long-term outcomes to LR for lesions under 3 cm. A multicenter retrospective analysis of patients with solitary CRLM (<3 cm) found that those undergoing MWA were older, had higher comorbidity scores, and more frequently had tumors in posterosuperior segments. Despite these differences, 5-year overall survival (51.5% vs 56.7%, *P* = 0.64) and disease-free survival (30.5% vs 36.2%, *P* = 0.10) were similar between MWA and LR, with a significantly shorter hospital stay for MWA (1 vs 4 days, *P* < 0.0001)^[^1^]^. These findings support MWA as a safe and effective option for carefully selected patients.

Several studies have reported durable local control for small colorectal liver metastases treated with microwave ablation (MWA), particularly when adequate ablation margins are achieved under precise imaging guidance^[^2^]^. Tumor size, anatomical location, and operator experience are critical factors influencing recurrence and overall outcomes^[^3^]^. Meta-analyses further demonstrate that MWA is feasible and effective for tumors located in posterosuperior liver segments, which are often technically challenging to access surgically^[^4^]^.These findings collectively highlight the importance of careful patient selection, meticulous procedural planning, and adherence to standardized ablation protocols to optimize long-term oncologic results.

While promising, several considerations remain. LR continues to provide optimal oncologic clearance for fit patients and may be preferred when multiple lesions or high-risk features are present. MWA outcomes are operator-dependent, and standardization of technique is essential. Most evidence, including multicenter studies, is retrospective; prospective multicenter trials are needed to confirm noninferiority, refine indications, and integrate MWA with systemic therapies. Cost-effectiveness analyses are also limited, particularly in resource-constrained settings.

Clinically, MWA represents a safe, effective, and minimally invasive alternative for patients with small solitary CRLM, particularly older or comorbid patients and those with tumors in technically challenging segments. Multidisciplinary evaluation, careful patient selection, and adherence to technical best practices are critical to achieving optimal outcomes.

In conclusion, multicenter evidence supports the integration of laparoscopic MWA alongside surgical resection for small solitary CRLM. Future research, including standardized protocols and prospective trials, is essential to further define indications, optimize technique, and ensure long-term oncologic efficacy. The letter to the editor follows the guidance of the Transparency in the Reporting of Artificial Intelligence in Research (TITAN) guideline^[^5^]^.

## Data Availability

Not applicable.
